# Supplementary material for: Obesity Stigma: Is the ‘Food Addiction’ Label Feeding the Problem?
Source: Nutrients. 2019 Sep 4;11(9):2100. doi: 10.3390/nu11092100 (PMC6770691; doi:10.3390/nu11092100)
Supplement: Supplementary file 1 [file nutrients-11-02100-s001.docx]

Supplementary material: Obesity stigma: Is the ‘food addiction’ label feeding the problem?

Helen K. Ruddock^1,2^, Michael Orwin^1,3^, Emma J. Boyland^1^, Elizabeth H. Evans^3^, and Charlotte A. Hardman^1^*

^1^ Department of Psychological Sciences, University of Liverpool, UK; E-mails: [charlotte.hardman@Liverpool.ac.uk](mailto:charlotte.hardman@Liverpool.ac.uk); [e.boyland@liverpool.ac.uk](mailto:e.boyland@liverpool.ac.uk)

^2^ School of Psychology, University of Birmingham, UK; [Email:](mailto:Email:) [ruddochk@bham.ac.uk](mailto:ruddochk@bham.ac.uk)

^3^  School of Psychology, Newcastle University, UK; e-mails: [Elizabeth.evans@ncl.ac.uk](mailto:Elizabeth.evans@ncl.ac.uk)

***** Correspondence: charlotte.hardman@liverpool.ac.uk

1. Vignette

Picture of target (Paulina) and description provided in each condition.


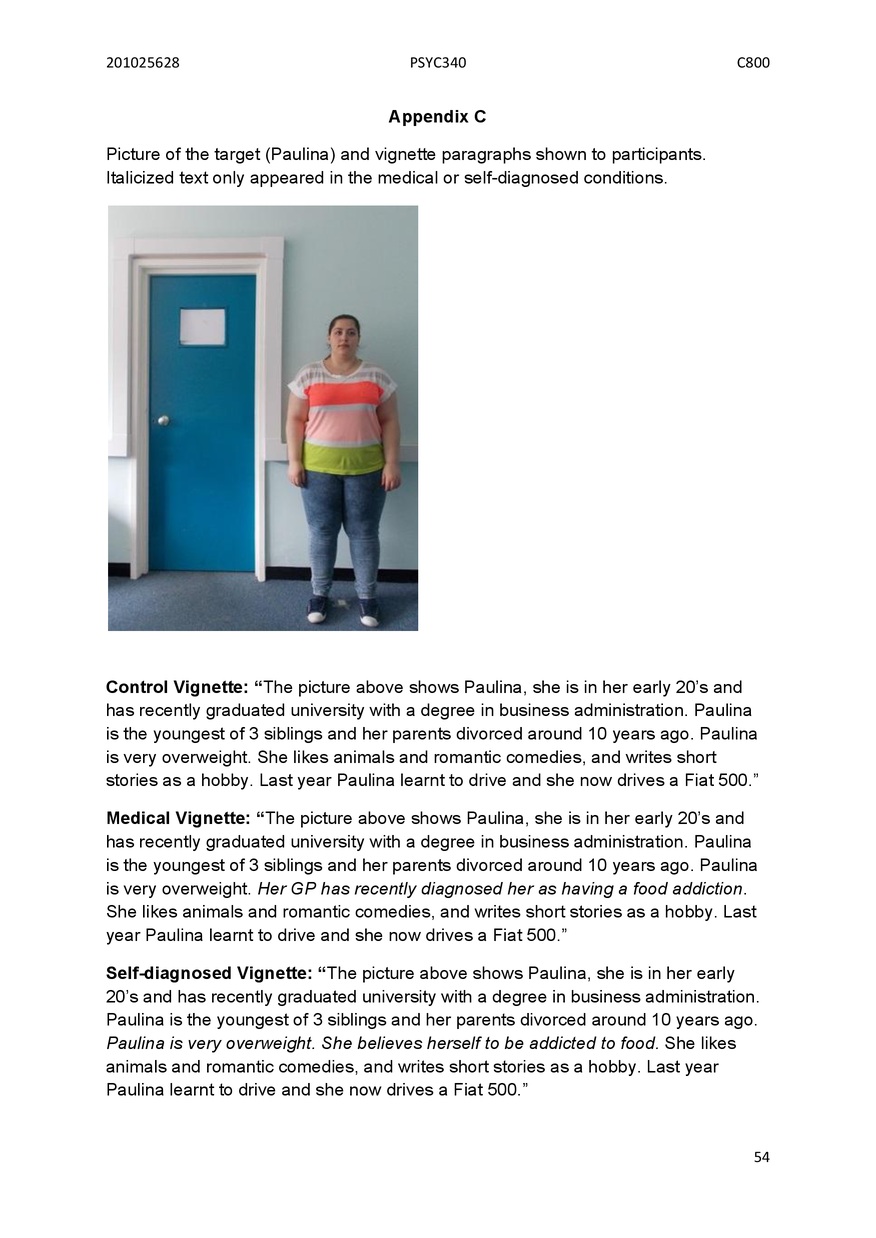


Control vignette

The picture above shows Paulina, she is in her early 20’s and has recently graduated university with a degree in business administration. Paulina is the youngest of 3 siblings and her parents divorced around 10 years ago. Paulina is very overweight. She likes animals and romantic comedies, and writes short stories as a hobby. Last year Paulina learnt to drive and she now drives a Fiat 500.

Self-diagnosed vignette

The picture above shows Paulina, she is in her early 20’s and has recently graduated university with a degree in business administration. Paulina is the youngest of 3 siblings and her parents divorced around 10 years ago. Paulina is very overweight. *She believes herself to be addicted to food*. She likes animals and romantic comedies, and writes short stories as a hobby. Last year Paulina learnt to drive and she now drives a Fiat 500.

Medical vignette

The picture above shows Paulina, she is in her early 20’s and has recently graduated university with a degree in business administration. Paulina is the youngest of 3 siblings and her parents divorced around 10 years ago. Paulina is very overweight. *Her GP has recently diagnosed her as having a food addiction.* She likes animals and romantic comedies, and writes short stories as a hobby. Last year Paulina learnt to drive and she now drives a Fiat 500.

2. Exploratory analyses

Do employability ratings differ as a function of condition in Studies 1 and 2?

For each participant we calculated the mean employability rating across all seven items. Univariate ANOVAs were conducted to examine whether employability ratings differed as a function of condition in studies 1 and 2. Mean employability ratings were entered as the dependent variable, and condition (i.e. Control, Medical, Self-diagnosed) was entered as the independent variable. In both studies, condition elicited no significant effect on employability ratings (Study 1: F(2, 437)=.914, p=.402; Study 2: F(2, 520)=2.77, p=.064).

2.1. Does student status moderate the effect of condition on general stigma (i.e. anti-fat attitudes, AFA) or target-specific stigma (i.e. Modified fat-phobia scale, M-FPS)?

Linear regression models were conducted to examine whether participants’ student status (i.e. student vs. non-student) moderated the effect of condition on AFA and M-FPS scores in Study 2. For each model, student status and condition (dummy coded, i.e. self-diagnosed vs. control / medical vs. control) were entered into Step 1. Interaction terms (i.e. student status x Self-diagnosed (vs. control) and student status x Medical condition (vs. control)) were included in Step 2. AFA and M-FPS scores were entered as the dependent variable in each model.

2.1.1. Target-specific stigma (M-FPS)

Student status significantly predicted M-FPS scores in Step 1 of the model such that *non*-students scored lower on this measure than students, B=-.051, SE=.041, t=-3.63, p<.001. However, in Step 2 of the model, there was no interaction between student status and self-diagnosed (vs. control) condition on M-FPS scores (B= -.110 , SE=.102, t=-1.09, p=.278), or between student status and medical (vs. control) condition (B=.094, SE=.101, t=.929, p=.353).

2.1.2. General stigma (AFA)

Student status significantly predicted AFA scores in Step 1 of the model. Non-students scored lower on this measure than students, B= -.328, SE=.09, t=-3.67, p<.001. In Step 2 of the model, there was no interaction between student status and self-diagnosed (vs. control) condition on AFA-scores (B= .039 , SE=.22, t=-.178, p=.859), or between student status and medical (vs. control) condition (B=-.031, SE=.22, t=-.142, p=.887).

2.2. Does participants’ age moderate the effect of condition on general stigma (AFA) or target-specific stigma (M-FPS)?

Heirarchical linear regression models were conducted to examine whether age moderated the effect of condition on AFA and M-FPS scores in Study 1 and 2. Age (centered), and condition (dummy coded) were entered in Step 1 of the model and the age x condition interaction terms were included in Step 2.

2.2.1 Study 1

Target specific stigma

Age did not significantly predict M-FPS scores in Step 1 or Step 2 of the model (Step 1: B=-.004, SE=.003, t=1.08, p=.283; Step 2: B=-.001, SE=.005, t=-.100, p=.920). In Step 2 of the model, age did not significantly moderate the effect of the medical condtiion (B=.000, SE=.008, t=.001, p=.999) or self-diagnosed condition (B=-.008, SE=.008, t=1.056, p=.291) on M-FPS scores.

General stigma

Age did not significantly predict AFA scores in Step 1 or Step 2 of the model (Step 1: B=-.004, SE=.004, t=-1.068, p=.286; Step 2: B=-.006, SE=.006, t=-990, p=.323). Age also did not moderate the effect of the medical condition (B=.007, SE=.010, t=.771, p=.441) or self-diagnosed condition (B=.001, SE=.009, t=.057, p=.955) on AFA scores.

2.2.2. Study 2

Linear regression models were conducted to examine whether participants’ age moderated the effect of condition on AFA and M-FPS scores in Study 2. For each model, age (centered), and dummy-coded conditions (i.e. Self-diagnosed vs. Control and Medical vs. Control) were entered into Step 1. Interaction terms (i.e. age x Self-diagnosed (vs. Control) and age x Medical condition (vs. control)) were included in Step 2. AFA and M-FPS scores were entered as the dependent variable in each model.

Target specific stigma (M-FPS)

Age negatively predicted M-FPS scores in Step 1 and Step 2 of the model (Step 1: B=-.006, SE=.00, t=-3.25, p=.001; Step 2: B=-.007, SE=.003, t=-2.38, p=.018). However, there was no significant interaction between age and condition on M-FPS scores (age x Medical vs. Control: B=.007, SE=.00, t=1.67, p=.096; age x Self-diagnosed vs. Control condition: B=-.004, SE=.00, t=-.858, p=.391).

General stigma (AFA)

Age negatively predicted AFA scores in Step 1 and Step 2 of the model (Step 1: B=-.018, SE=.00, t=-4.70, p<.001; Step 2: B=-.018, SE=.01, t=2.81, p=.005). There was no significant interaction between age and Medical (vs. Control) or Self-diagnosed (vs. Control) condition on AFA scores, (age x Medical vs. Control: B=-.006, SE=.01, t=.603, p=.547; age vs. Self-diagnosed vs. Control: B=.005, SE=.01, t=.523, p=.601).

2.3. Do DEBQ scores moderate the effect of condition on general stigma (AFA) or target-specific stigma (M-FPS)?

Hierarchical linear regression models were conducted to examine whether DEBQ restraint, emotional eating, and external eating scores (centered) moderated the effect of condition on AFA or M-FPS scores. For each model, DEBQ subscale scores and condition (dummy coded) were entered into Step 1, and interaction terms were entered into Step 2.

2.3.1. Study 1

Restrained eating

DEBQ-restraint scores significantly and positively predicted AFA scores in Step 1 and Step 2 of the model (Step 1: B=.215, SE=.028, t=7.803, p<.001; Step 2: B=.210, SE=.050, t=4.199, p<.001). Restraint scores did not moderate the effect of condition on AFA scores (Medical condition: B=-.001, SE=.067, t=-.009, p=.993; Self-diagnosed condition: B=.016, SE=.070, t=.224, p=.823).

DEBQ-restraint scores significantly and positively predicted M-FPS scores in Step 1 and Step 2 of the model (Step 1: B=.107, SE=.025, t=4.329, p<.001; Step 2: B=.136, SE=.045, t=3.023, p=.003). The DEBQ x condition interaction terms did not predict M-FPS scores (Medical condition: B=-.013, SE=.060, t= -.221, p=.825; Self-diagnosed condition: B=-.074, SE=.063, t=1.180, p=.239).

Emotional eating

DEBQ-emotional eating scores significantly and positively predicted AFA scores in Step 1 and Step 2 of the model (Step 1: B=.093, SE=030, t=3.099, p=.002; Step 2: B=.137, SE=.053, t=2.571, p=.010). The condition x DEBQ-emotional eating interaction terms did not significantly predict AFA scores (Medical: B=-.132, SE=.073, t=1.798, p=.073; Self-diagnosed: B=.006, SE=.074, t=.086, p=.931).

DEBQ-emotional eating scores significantly and positively predicted M-FPS scores in Step 1 of the model, but this was not significant in Step 2 (Step 1: B=.056, SE=.026, t=2.154, p=.032; Step 2: B=.079, SE=.046, t=1.72, p=.086). The DEBQ-emotion x condition interaction terms did not significantly predict M-FPS scores (Medical: B=.009, SE=.064, t=.142, p=.887; Self-diagnosed: B=-.061, SE=.064, t=-.946, p=.345).

External eating

DEBQ-external eating scores did not predict AFA scores in Step 1 or Step 2 of the model (Step 1: B=.034, SE=.040, t=.848, p=.397; Step 2: B=.042, SE=.066, t=.634, p=.526). The DEBQ-external x condition interaction terms were also not significant (Medical: B=-.054, SE=.094, t=-.579, p=.563; Self-diagnosed: B=.047, SE=.103, t=.454, p=.650).

DEBQ-external eating scores positively predicted M-FPS scores in Step 1, but not in Step 2, of the model (Step 1: B=.109, SE=.034, t=3.192, p=.002; Step 2: B=.089, SE=.056, t=1.60, p=.110). M-FPS scores were not predicted by the DEBQ-external eating x condition interaction terms (Medical: B=.098, SE=.079, t=1.23, p=.219; Self-diagnosed: B=-.060, SE=.087, t=-.690, p=.491).

2.3.2. Study 2

Restrained eating

Restraint siginficantly and positively predicted AFA scores in Step 1 and Step 2 of the model (Step 1: B=.410, SE=.048, t=8.592, p<.001; Step 2: B=.333, SE=083, t=4.015, p<.001. The restraint x condition interaction terms did not significantly predict AFA scores (Medical: B=.107, SE=.116, t=.925, p=.355; Self-diganosed: B=.124, SE=.119, t=1.043, p=297).

Restraint scores significanlty and positively predicted M-FPS scores in Step 1, but not in Step 2 of the model (Step 1: B=091, SE=.023, t=3.93, p<.0001; Step 2: .039, SE=040, t=.959, p=.338). M-FPS scores were not predicted by the condition x restraint interaction terms (Medical: B=.109, SE=.056, t=1.946, p=.052; Self-diagnosed: B=.045, SE=.058, t=.779, p=.437).

Emotional eating

DEBQ-emotional eating scores siginficantly and positively predicted AFA scores in Step 1, but not Step 2, of the model (Step 1: B=.164, SE=.047, t=3.499, p=.001; Step 2: B=.116, SE=.080, t=1.446, p=.149). The emotional x condition interaction terms did not significantly predict AFA scores (Medical: B=.041, SE=.117, t=.351, p=.726; Self-diganosed: B=.102, SE=.112, t=.905, p=.366).

DEBQ-emotional eating scores did not significanlty predict M-FPS scores in Step 1 or Step 2 of the model (Step 1: B=.009, SE=.022, t=.400, p=.689; Step 2: B=.011, SE=.037, t=.299, p=.765). M-FPS scores were not predicted by the condition x emotional interaction terms (Medical: B=-.024, SE=.055, t=-.434, p=.664 ; Self-diagnosed: B=.013, SE=.052, t=.255, p=.799).

External eating

DEBQ-external eating scores significantly and positively predicted AFA scores in Step 1 and Step 2 (Step 1: B=.315, SE=.078, t=4.038, p<.001; Step 2: B=.297, SE=.141, t=2.104, p=.036). AFA scores were not significantly predicted by the condition x external eating interaction terms (Medical: B=-.030, SE=.193, t=-.157, p=.875; Self-diagnosed: B=.086, SE=.195, t=.442, p=.658).

DEBQ-external eating scores significantly and positively predicted M-FPS scores in Step 1, but not Step 2, of the model (Step 1: B=.078, SE=.036, t=2.132, p=.033; Step 2: B=.097, SE=.066, t=1.468, p=.143). The condition x external eating interaction terms did not significantly predict M-FPS scores (Medical: B=-.020, SE=.090, t=-.223, p=.824; Self-diagnosed: B=-.035, SE=.091, t=-.389, p=.698).

*2.4. Gender differences on AFA subscales (Study 2 only)*

We explored whether males and females differed on each subscale of the AFA (i.e. ‘dislike’, ‘fear’, ‘willpower’). We entered all three subscales into a MANOVA with gender as the independent variable. This revealed significant differences between males and females on the AFA subscales, F(3,519)=20.91, p<.001, ηp²=.108. Males scored significantly higher on the ‘dislike’ subscale (Males: Mean=3.20, SE=.09; Females: M=2.90, SE=.067) F(1,521)=7.41, p=.007, ηp²=.014, and the ‘willpower’ subscale (Males: Mean=6.21, SE=.10; Females: Mean=5.88, SE=.07), F(1,521)=7.86, p=.005, ηp²=.015. Females scored higher on the ‘fear’ subscale, F(1,521)=36.44, p<.001, ηp²=.065 (Males: M=4.80, SE=.14; Females: M=5.84, SE=.07). Results thus suggest that males have greater dislike towards people with obesity, and hold the belief that obesity is caused by a lack of willpower, while females have a greater fear of personal weight gain.

To explore whether gender moderated any effect of condition on AFA subscales, we performed 2 (gender) x 3 (condition) ANOVAs with ‘fear’, ‘willpower’, and ‘dislike’ subscales. There was a gender x condition interaction on ‘willpower’ subscale scores, F(2,517)=3.17, p=.043, ηp²=.012 (Figure S1). There was no gender x condition interaction for ‘dislike’ subscale scores, F(2,517)=.901, p=.407, ηp²=.003, or on ‘fear’ subscale scores, F(2,517)=2.61, p=.075, ηp²=.010. Pairwise comparisons revealed that in the control condition, males had significantly higher AFA-willpower scores than females (p<.001) (Males: M=6.44, SE = .17; Females: M=5.69, SE=.13). However, AFA willpower did not differ between males and females in self-diagnosed (p=.869) and medical conditions (p=.330) (Figure S1).

2.5.. Gender differences on age, BMI, and eating behaviour (Study 2 only)

A MANOVA was conducted to explore whether males and females differ with regards to age, BMI, and scores on measures of eating behaviour (i.e. DEBQ). This revealed significant gender differences, F(5, 511)=18.51, p<.001, ηp²=.153 (Table S1).

Table S1. Participant characteristics as a function of gender in Study 2. Results are means (standard deviations) unless otherwise specified (**significant difference, p<.01).

| **Variable** | **Males (n=192)** | **Females (n=325)** | **Between-group differences** |
| --- | --- | --- | --- |
| Age (y) | 25.94 (±9.81) | 27.79 (±12.12) | F(1,515)=3.37, p=.066 |
| BMI (kg/m^2^) | 24.23 (±3.96) | 23.18 (±4.21) | F(1,515)=7.78, p=.005** |
| DEBQ-Restraint | 2.40 (±0.81) | 2.88 (±0.88) | F(1,515)=38.98, p<.001** |
| DEBQ-Emotion | 2.41 (±0.88) | 2.86 (±0.96) | F(1,515)=27.48, p<.001** |
| DEBQ-External | 3.31 (±0.57) | 3.31 (±0.57) | F(1,515)= 0.00, p=.996 |

*b*

*a*

*Figure S1.* Scores on AFA-Willpower subscale as a function of condition and gender in Study 2. Different letters (e.g. *a,b)* indicate significant differences.
